# No evidence of extensive non-CpG methylation in mtDNA

**DOI:** 10.1093/nar/gkac701

**Published:** 2022-08-18

**Authors:** Romain Guitton, Gonzalo S Nido, Charalampos Tzoulis

**Affiliations:** Neuro-SysMed, Department of Neurology, Haukeland University Hospital, 5021 Bergen, Norway; Department of Clinical Medicine, University of Bergen, Pb 7804, 5020 Bergen, Norway; Neuro-SysMed, Department of Neurology, Haukeland University Hospital, 5021 Bergen, Norway; Department of Clinical Medicine, University of Bergen, Pb 7804, 5020 Bergen, Norway; Neuro-SysMed, Department of Neurology, Haukeland University Hospital, 5021 Bergen, Norway; Department of Clinical Medicine, University of Bergen, Pb 7804, 5020 Bergen, Norway

## Abstract

While most research suggests mitochondrial DNA (mtDNA) harbors low or no methylation, a few studies claim to report evidence of high-level methylation in the mtDNA. The reasons behind these contradictory results are likely to be methodological but remain largely unexplored. Here, we critically reanalyzed a recent study by Patil *et al.* (2019) reporting extensive methylation in human mtDNA in a non-CpG context. Our analyses refute the original findings and show that these do not reflect the biology of the tested samples, but rather stem from a combination of methodological and technical pitfalls. The authors employ an oversimplified model that defines as *methylated* all reference positions with methylation proportions above an arbitrary cutoff of 9%. This substantially exacerbates the overestimation of methylated cytosines due to the selective degradation of unmethylated cytosine-rich regions. Additional limitations are the small sample sizes and lack of sample-specific controls for bisulfite conversion efficiency. In conclusion, using the same dataset employed in the original study by Patil *et al.*, we find no evidence supporting the existence of extensive non-CpG methylation in the human mtDNA.

## INTRODUCTION

Mitochondrial DNA (mtDNA) is a 16 569 bp long, circular DNA molecule, which is inherited from the mother, and is present in multiple copies per mitochondrion and cell. mtDNA comprises 37 genes encoding 13 peptide subunits of the respiratory chain, as well as the 22 tRNAs and two rRNAs required for intra-mitochondrial translation. The two strands of mtDNA have been termed heavy (H) and light (L) strands due to their different purine/pyrimidine content. While little is known about the epigenetic control of the mitochondrial genome, cytosine methylation has been postulated as a candidate mechanism for epigenetic regulation of its expression. However, the existence of methylation in the mammalian mtDNA remains the subject of debate (1–4). Given the central role of mtDNA in cellular metabolism and the potential function of mtDNA methylation may have in controlling its expression, this is an important controversy to settle.

Currently, bisulfite (BS) reduction of DNA followed by high-throughput sequencing is considered the gold standard to assess cytosine (C) methylation at single-base resolution (5,6). Treating DNA with BS results in the specific conversion of unmethylated Cs into uracil, allowing to measure methylation levels by comparing to the unconverted reference. During the harsh treatment, however, DNA is also exposed to degradation by BS. The degree of degradation occurring in mtDNA samples has consistently been shown to be more severe in the L strand due to the relatively higher C content, resulting in a strong strand coverage bias (3,4,7,8). Furthermore, BS-induced degradation preferentially affects unmethylated Cs in the sample, resulting in overestimation of methylation levels in C-rich regions (9).

While most recent studies report either very low or no methylation in mtDNA (2–4,8,10–12), including BS-independent methods capable of native methylation detection (13,14), at least three studies contradict this conclusion (15–17). Prominent among them, the work by Patil *et al.* (1) claims to provide evidence that the human mtDNA is extensively methylated in a non-CpG context. Intrigued by the conspicuous discrepancy between these and other findings, we critically reanalyzed the data presented in Patil *et al.* Reanalysis of the dataset failed to reproduce the reported extensive mtDNA methylation, thus refuting the main conclusion of the study. Furthermore, we show that the high levels of mtDNA methylation reported in the original study resulted from methodological pitfalls. Specifically, the original findings are based on an oversimplified model that defines as *methylated* all reference positions with methylation proportions above an arbitrary cutoff of 9%. This approach greatly exacerbates the overestimation of methylated cytosines due to selective degradation of unmethylated cytosine-rich regions.

## MATERIALS AND METHODS

Original FASTQ files were kindly provided by the authors (1) (Table 1). With the exception of a single corrupted file, (Lib6_S5) all samples were aligned using Bismark v0.22.3 (18). Due to the existence of regions of homology between mtDNA and nuclear DNA, alignment of BS-sequencing data against the whole genome typically results in a considerable loss of mitochondrial reads to the nuclear genome. In contrast, the rate of nuclear read misalignment onto the mtDNA reference is negligible (12). For this reason, alignment was performed against the rCRS mitochondrial reference. Assessment of the resulting M-plots and QC reports of FastQC (19) guided the choice of trimming parameters, and FASTQ files were trimmed using Trimmomatic v0.39 (20) with the following settings: ILLUMINACLIP:TruSeq3-PE.fa:2:30:10 CROP:75 HEADCROP:7 LEADING:3 TRAILING:3 SLIDINGWINDOW:4:15 MINLEN:15. Results from M-bias estimation (data and plots) are available in the Appendix of the associated GitLab repository. Trimmed reads were re-aligned against the rCRS reference, deduplicated, and methylation extracted using the Bismark suite. Samples were also aligned against the lambda phage reference (GenBank J02459.1) to test for the presence of spike-ins in the samples. Nucleotide coverage bias was calculated using the bam2nuc script from the Bismark suite.

**Table 1.** Number of reads aligned to the mtDNA H- and L-strands, coverage strand bias (H-strand/L-strand) and C coverage bias (observed/expected C coverage) for Patil *et al.*

LibID	SampleID	H-strand	L-strand	H/L	C cov. bias
Lib9_S3	HepaRG_D0	13 623 164	1 685 526	8.08	0.50
Lib12_S5	Hepatocytes	57 677 545	5 260 406	10.96	0.50
HepG2_BamH1	HepG2_BamH1	77 064 281	32 301 489	2.39	0.61
Lib1_S1	HepG2_Lib1	62 919 874	49 634 149	1.27	0.68
Lib2_S2	HepG2_Lib2	31 539 298	809 697	38.95	0.41
Lib10_S4	HMEC	21 918 410	10 658 456	2.06	0.63
Lib5_S4	MCF10A	33 781 446	796 892	42.39	0.41
Lib6_S5	MCF10A_DNMT1_KO_siRNA (FAILED)	NA	NA	NA	NA
LIb7_S6	MCF10A_DNMT3A KO_siRNA	26 788 362	2 590 917	10.34	0.49
LIb8_S7	MCF10A_DNMT3B_K0_siRNA	11 167 166	357 647	31.22	0.42
MCF7	MCF7	78 125 988	15 055 644	5.19	0.54
Lib11_S2	MCF7_Lib11	128 959 283	19 707 971	6.54	0.53
NormalLiver	Normal_liver	3 649 371	691 199	5.28	0.51
HepaRG	Normal_liver_2	13 623 164	1 685 526	8.08	0.50
Lib4_S3	Primary_tumour_liver_HCC	10 731 892	409 268	26.22	0.43
Lib8_S2	Lambda	23 133	4 926	4.70	1.03

All subsequent downstream analyses were conducted using the R scripting language and are available in their entirety in a publicly available GitLab repository. The code deposited in this repository allows to reproduce the results discussed in this letter in their entirety.

## RESULTS AND DISCUSSION

**Figure 1.**
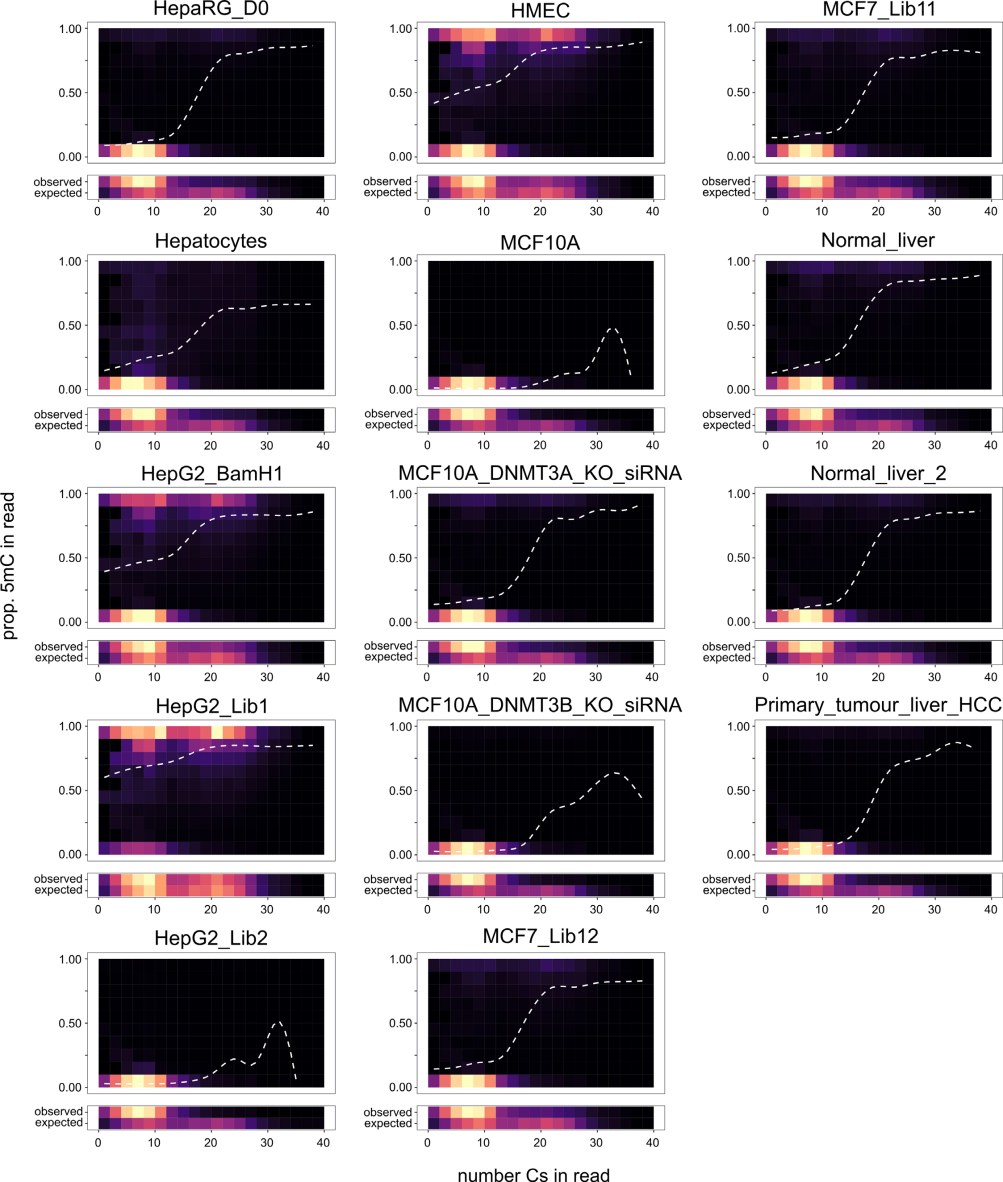
Methylation overestimation prior to sequencing. Each single sequencing read bears information from contiguous positions from the same DNA molecule. While this information is generally discarded by analyzing the aggregated alignment data, it can be effectively leveraged to extract valuable information about the nature of the sample. The plots represent each sequencing read according to (x-axis) its total number of reference cytosines (i.e. an indication of how C-rich the mtDNA region was), and (y-axis) the proportion of those Cs that are methylated. Hence, the x-axis represents the mtDNA reference in terms of its relative C-content, with C-poor mtDNA segments on the left-hand side of the panels and C-rich on the right-hand side. The y-axis, on the other hand, represents the local methylation levels. To avoid cluttering, points (sequencing reads) are summarized in density tiles, with lighter shades indicating higher densities of points (relative to each sample/plot). A smoothed estimate (dashed curve) summarizes the main trend. Additionally, at the bottom of each plot, we represent the marginal distribution for the observed x-axis values (‘observed’) compared with the ‘expected’ distribution in a uniform mtDNA coverage. Despite notable differences across samples, a trend emerges – reads originating from C-poor mtDNA regions are mostly fully unmethylated (and overrepresented) while reads originating from C-rich mtDNA regions tend to be fully methylated and are underrepresented compared to the theoretical distribution in a uniformly covered mtDNA (as illustrated by the bottom marginal distribution plots). Such a trend is expected from the exclusion of unmethylated C-rich fragments prior to amplification, where the few C-rich fragments left are protected from degradation by 5mC (9).

In light of previous evidence suggesting that linearization of the circular mtDNA prior to bisulfite (BS) treatment may be essential for increasing BS conversion efficiency (2,7,8,21), Patil *et al.* first compare a strategy of enzymatic mtDNA linearization to sonication prior to BS conversion. Following this comparison, which is carried out in a single sample (HepG2), they conclude that the sonication approach is superior because it results in a lower heavy (H) over light (L) mtDNA strand coverage bias and better overall mtDNA coverage. Specifically, the authors claim that they observe a reduction in strand coverage bias (H/L) from 0.08 with enzymatic linearization, to 1.16 with sonication. Reanalysis of the data did not recapitulate the reported strand bias of 0.08, and showed, instead, a bias of 2.39, with the expected overrepresentation of reads from the H strand (Table 2), as has been consistently reported before (9) Hence, while we also observe a lower strand coverage bias with the sonication approach (H/L: 1.27), compared to the linearization approach (H/L: 2.39), the mitigation is of a much lesser magnitude than what the authors report. More importantly, our analyses detected much larger coverage bias across the other samples analyzed in the study, which had been processed by sonication only, with values of up to H/L: 42 (Table 1 and Figure 1). Given this high variability across samples and since linearization data is only available from a single sample, no statistical support can be provided on the effect of the two approaches with respect to strand bias.

The authors go on to compare the percentage of unconverted Cs relative to the depth of coverage between the linearized and sonicated HepG2 sample (Figure 1B and C in the original Patil *et al.* publication). While the range of coverage is comparable in the two preparations, the unconverted methylation percentages are alarmingly different. The linearized preparation shows overall very low mtDNA methylation levels, whereas the sonicated preparation exhibits high levels of mtDNA methylation. If, as the authors claim, there was widespread mtDNA methylation in the samples, the low methylation levels shown for the linearized sample in Figure 1B could only be explained by a massive conversion of 5mC bases, which is both unrealistic and would signify that BS conversion can no longer be used to assess methylation levels. On the other hand, if we assume a general lack of mtDNA methylation in the samples, these results would be highly compatible with: (i) an inefficient BS conversion in the sonicated sample, a known limitation of this technique, and (ii) the known exclusion of unmethylated C-rich fragments prior to amplification, a phenomenon known to be particularly severe with high-temperature denaturation kits, such as the ones employed by the authors (9). Despite the latter scenario providing a much more parsimonious interpretation, the authors assume the existence of high methylation levels in the sample and choose to regard sonication as a better method for BS library preparation. This conclusion is unfounded since current evidence indicates that: (i) linearization alleviates BS conversion inefficiency (7,8,11) and (ii) the use of additional sonication stages in library preparation of BS samples results in a selective degradation of C-rich unmethylated DNA, effectively overestimating methylation levels and worsening coverage- and methylation H/L strand biases (9). In summary, the results of Patil *et al.* do not support the notion that a sonication step for mtDNA methylation analysis prior to BS treatment is advisable in place of enzymatic linearization or in addition to it.

An important limitation in the work by Patil *et al.* is that the BS conversion rate is not adequately controlled. When performing BS sequencing, it is essential to include unmethylated controls as spike-ins into the samples in order to estimate the efficiency of conversion for each sample independently. For reasons unclear in the paper, the authors did not include spike-ins with every sample. The authors, instead, carried out an independent BS sequencing run of an unmethylated lambda phage DNA. Based on the background level of non-converted Cs in this single unmethylated control, the authors calculated the false positive rate (FPR) of 5mC calls. However, the premises used for this calculation are flawed. They defined their FPR as the ratio between the number of 5mCs over the total number of Cs in the lambda phage DNA:

$$\begin{equation*} FPR = \left[ {\left( {\frac{{\#\,methylated\,cytosines\,in\,plasmid\,genome}}{{total\,\# \,cytosines\,in\,plasmid\,genome}}} \right)} \right] \end{equation*}$$

The FPR that the authors employ refers to genomic positions rather to sequencing basecalls. In this context, any given C position in the reference genome can only take one of two values, either *methylated* or *unmethylated*. This is an oversimplification which does not reflect the complexity of the sample. In reality, each C may exhibit a methylation proportion ranging anywhere between 0–100%. This is due to: (i) technical factors, i.e. the efficiency of BS conversion is never 100% and non-converted Cs are subject to subsequent amplification prior to sequencing, and (ii) biological samples are made up of a heterogeneous population of different cell types and/or cells in different stages, which exhibit different methylation profiles. This is especially relevant in the case of mtDNA, which exists in multiple copies per cell and commonly exhibits heteroplasmic variation.

The authors use the FPR to estimate an *accuracy* (ACC = 1 – FPR), where they define as *methylated* (i.e. false positive, FP) each C position in the lambda genome with a methylation of 9% or more and a coverage of at least 10×, and as *unmethylated* (or ‘discarded’) each position with a methylation below 9% or 10x coverage. The authors argue that the choice of these specific parameters for each of the other samples ensured that the methylation calls from the data are at least 98.95% accurate. This claim is incorrect since their accuracy measure is based solely on an unmethylated sample, and hence does not contemplate true positives (i.e. rightly called 5mCs) nor false negatives (i.e. wrongly called unmethylated Cs). Thus, this approach by Patil *et al.* only controls the FPR (type I error). In addition, within this framework, a hypothetical sample with a 9% methylation proportion across all Cs will be considered 100% methylated, whereas a sample with an 8.9% methylation proportion across all Cs will be considered 100% unmethylated. Even more concerning, all Cs with a coverage below 10× will be considered *unmethylated* instead of simply being discarded from the analysis due to the lack of sufficient sampling. The proportion of unmethylated genome reported by the authors (represented in the original publication in Figures 2C and 3C for a total of four samples) corresponds, therefore, to the combination of: (i) methylation levels below 9% and (ii) positions of low coverage (including zero coverage). This oversimplified methylation model is especially problematic considering the selective degradation of unmethylated C-rich DNA that is known to occur with BS library preparation, most severely so with the high-temperature denaturation kits used by the authors (9). This bias was in fact evident in all reanalyzed samples (Figure 1), and results in a generalized overestimation of methylation—further exacerbated by the arbitrary definition of positions showing over 9% methylation proportion as fully methylated.

Finally, to complement the BS sequencing findings, the authors claim that MeDIP-seq on two samples (two replicates each) showed that the mitochondrial genome was globally methylated. Such claim, however, cannot be made using a relative (within-sample) quantification technique such as MeDIP-seq, since the same result can be obtained by two samples with dramatic differences in methylation levels as long as they are uniform (e.g. 5% methylation in all Cs versus 100% methylation in all Cs).

In conclusion, while our reanalysis does not provide proof of the absence of methylation in mtDNA, we prove that the results by Patil *et al.* claiming a widespread mtDNA methylation are invalid and driven by fundamental methodological flaws, rather than reflecting the biology of the tested samples. An oversimplified definition of methylation combined with a choice of library preparation known to overestimate methylation levels are responsible for the erroneous conclusions of the study. In conjunction with the lack of appropriate sample-specific controls, the data presented by Patil *et al.* cannot be used to reliably assess mtDNA methylation and should at best be considered non-informative. In line with this conclusion, the majority of studies on this subject indicate the mtDNA is either unmethylated or harbors low methylation levels in human tissue (3,8,10–12). This is also supported by newer studies which employ native methylation detection and are, hence independent of bisulfite conversion of DNA (13,14).

## DATA AVAILABILITY

All analyses were conducted using the R scripting language and are available in their entirety in a publicly available GitLab repository: https://git.app.uib.no/neuromics/reanalysis-patil-et-al.
